# Human Papillomavirus Vaccination in Male and Female Adolescents Before and After Kidney Transplantation: A Pediatric Nephrology Research Consortium Study

**DOI:** 10.3389/fped.2020.00046

**Published:** 2020-02-20

**Authors:** Corina Nailescu, Raoul D. Nelson, Priya S. Verghese, Katherine E. Twombley, Aftab S. Chishti, Michele Mills, John D. Mahan, James E. Slaven, Marcia L. Shew

**Affiliations:** ^1^Department of Pediatrics, Indiana University, Riley Hospital for Children, Indianapolis, IN, United States; ^2^Department of Pediatrics, University of Utah, Primary Children's Hospital, Salt Lake City, UT, United States; ^3^Department of Pediatrics, University of Minnesota Masonic Children's Hospital, Minneapolis, MN, United States; ^4^Department of Pediatrics, Medical University of South Carolina Children's Hospital, Charleston, SC, United States; ^5^Department of Pediatrics, Kentucky Children's Hospital, University of Kentucky, Lexington, KY, United States; ^6^Department of Pediatrics, C. S. Mott Children's Hospital, University of Michigan, Ann Arbor, MI, United States; ^7^Department of Pediatrics, Nationwide Children's Hospital, The Ohio State University, Columbus, OH, United States; ^8^Department of Biostatistics, Indiana University, Indianapolis, IN, United States

**Keywords:** HPV—human papillomavirus, vaccination, kidney transplantation, chronic kidney disease, dialysis, pediatric

## Abstract

**Background:** Kidney transplant (KT) recipients have higher incidence of malignancies, including Human Papillomavirus (HPV)-associated cancers. Thus, HPV vaccines may have an important role in preventing HPV-related disease in this population; however, immunogenicity and safety data are lacking.

**Objective:** To examine the immunological response and tolerability to HPV vaccination in pediatric KT recipients compared to future KT candidates.

**Methods:** The quadrivalent HPV vaccine was administered to girls and boys age 9–18 recruited from seven centers part of the Pediatric Nephrology Research Consortium. Subjects were recruited for three groups: (1) *CKD:* chronic kidney disease stages 3, 4, and 5 not on dialysis; (2) *Dialysis;* (3) *KT* recipients. The outcome consisted of antibody concentrations against HPV 6, 11, 16, and 18. Geometric mean titers (GMTs) and seroconversion rates were compared. Vaccine tolerability was assessed.

**Results:** Sixty-five participants were recruited: 18 in the CKD, 18 in the dialysis, and 29 into the *KT* groups. KT patients had significantly lower GMTs after vaccination for all serotypes. The percentages of subjects who reached seroconversion were overall lower for the *KT* group, reaching statistical significance for HPV 6, 11, and 18. Comparing immunosuppressed subjects (anyone taking immunosuppression medications, whether KT recipient or not) with the non-immunosuppressed participants, the former had significantly lower GMTs for all the HPV serotypes and lower seroconversion rates for HPV 6, 11, and 18. KT females had higher GMTs and seroconversion rates for certain serotypes. There were no adverse events in either group.

**Conclusions:** HPV vaccine was well-tolerated in this population. Pediatric KT recipients had in general lower GMTs and seroconversion rates compared to their peers with CKD or on dialysis. Immunosuppression played a role in the lack of seroconversion. Our results emphasize the importance of advocating for HPV vaccination prior to KT and acknowledge its safety post transplantation. Future studies are needed to investigate the effect of a supplemental dose of HPV vaccine in KT recipients who do not seroconvert and to evaluate the long-term persistence of antibodies post-KT.

## Introduction

Solid organ transplant (SOT) recipients are at increased risk for malignancies in both pediatric and adult populations (1–3), with an elevated risk of dying of their cancer, even after adjustment for stage and treatment (4). Pediatric and adult kidney transplant (KT) recipients carry similar risk and malignancies are third most common cause of mortality within this population (5, 6). Specifically, female KT recipients have an increased risk of cervical cancer up to 14-fold, vulvar cancer up to 50-fold and anal cancers up to 100-fold (7, 8). In addition, male SOT recipients have an increased risk for penile cancers (9). Human papillomavirus (HPV) 16 and to a much lesser extent HPV 18 are responsible for the majority of these cancers (10). In addition, HPV 16 is also responsible for a large proportion of oral pharyngeal cancers (11). Not only that HPV infections are the most prevalent sexually transmitted infections in the US (12), but non-carcinogenic HPV types, HPV 6 and 11, can be associated with genital warts and can be difficult to treat in the face of immunosuppression (13). Immunosuppressed individuals, including transplant recipients, have higher rates of HPV infections (14–16), greater likelihood of persistent infections (17, 18) and higher rates of HPV clinical disease, including cancer (9, 19, 20). Thus, preventing HPV infections in this population is critical. HPV-related vaccine trials have been shown to prevent HPV infection in immunocompetent individuals when the vaccine is given prior to viral exposure (21). In addition, more recent studies have demonstrated a true reduction in abnormal Pap smears and cervical cancers with use of HPV vaccine (22, 23). Because HPV is sexually transmitted and often acquired soon after the onset of sexual activity (24), vaccination should ideally occur before sexual debut.

The efficacy and safety of the HPV vaccine in certain immunocompromised populations, such as pediatric chronic kidney disease (CKD), dialysis and KT recipients, has not been well-studied. This is a population at increased risk for infections and HPV related sequelae; in addition, they appear to also be at high risk for not getting vaccinated due to lack of data and less immediate concerns about sexual activity when ill and awaiting transplant, as has been shown in other patients with severe chronic illnesses (25, 26). The goal of this study was to examine the immunological response and tolerability of the quadrivalent HPV vaccine in both female and male pediatric patients with CKD, on dialysis and with a KT. The primary hypothesis was that all these populations will mount an immune response to the vaccine. Our secondary hypothesis was that pediatric patients with CKD 3, 4, 5 and on dialysis, therefore future KT candidates, will respond to the HPV vaccination better than the KT recipients.

## Materials and Methods

### Study Population

Males and females ages 9–18 years were recruited from seven collaborating large medical centers in the Pediatric Nephrology Research Consortium: Riley Hospital for Children in Indianapolis, IN, Primary Children's Hospital in Salt Lake City, UT, Medical University of South Carolina Children's Hospital in Charleston, SC, University of Minnesota Masonic Children's Hospital in Minneapolis, MN, Kentucky Children's Hospital in Lexington, KY, C.S. Mott Children's Hospital in Ann Arbor, MI and Nationwide Children's Hospital in Columbus, OH. Subjects were recruited in three cohorts:

– Group 1 (CKD) included patients with CKD stages 3 [glomerular filtration rate (GFR) 30–59 mL/min per 1.73 m^2^], 4 (GFR 15–29 mL/min per 1.73 m^2^) or 5 not on dialysis (GFR <15 mL/min/1.73 m^2^, but not on dialysis).
– Group 2 (Dialysis) included patients on either chronic hemodialysis or peritoneal dialysis.
– Group 3 (KT) included patients who had received a KT at least 6 months prior to enrollment, in keeping with the recommendations of the American Society of Transplantation that inactivated vaccines may be resumed 3–6 months after a SOT (27).

Exclusion criteria were: pregnancy (self-reported), post-transplant lymphoproliferative disorder, active infectious diseases, fever, bleeding disorders, having received blood products in the past 6 months and hypersensitivity to any of the vaccine components.

### Study Design

All participants who met eligibility criteria and who had not been vaccinated against HPV were approached within the dialysis units or clinics by a study coordinator at each site. Study coordinators discussed the study and consent was obtained from the parent/guardian and assent from the participant. Medical records were used to abstract age, gender, race, and estimated GFR (eGFR) using the modified Schwartz formula (0.413 × height in cm/serum creatinine in mg/dL). For the CKD 3, 4, 5 and dialysis patients, it was documented whether they were on immunosuppression medications for their respective primary kidney diseases. The rationale for doing so was to perform sub-analyses to determine whether patients on immunosuppression medications (including KT recipients, but also patients with CKD 3, 4, and 5 taking immunosuppression medications to treat their primary disease) had a weaker immune response to the HPV vaccine compared to the rest. In addition, for KT patients, type of donor (living vs. deceased), number of transplants and time since most recent transplant were recorded. HPV quadrivalent vaccine (Gardasil®, Merck & Co., Inc.) was administered to each study participant per protocol according to the Advisory Committee on Immunization Practices recommendations at the time (28). Dose 1 of the HPV vaccine was administered to each study participant on day 1 of enrollment, after obtaining a baseline blood sample for antibody testing. Dose 2 was targeted to be administered at month 2 and dose 3 at month 6. The minimum interval between dose 1 and 2 of the vaccine was 4 weeks. The minimum recommended interval between dose 2 and 3 of the vaccine was 12 weeks. No subject received dose 2 or 3 more than 4 weeks later than initially targeted date. The Institutional Review Board of each participating center approved the study.

### Outcomes

The primary outcome was the antibody response to each of the four HPV serotypes contained within the vaccine (6, 11, 16, 18). Serum was collected from all subjects on day 1 of enrollment prior to dose 1 of the vaccine administration [Time 1 (T1)] and at month 7 (or 1 month after vaccine series was completed) [Time 2 (T2)]. Anti-HPV responses were measured using a competitive Luminex Immunoassay performed by PPD Vaccines and Biologics (Wayne, PA) and expressed as geometric mean titers (GMTs). A participant was considered to have seroconverted if no antibody titers were found at enrollment and antibody levels post immunization were above the sero-status cutoffs for HPV types 6, 11, 16, and 18, as determined by the assay developer (GMTs ≥ 20, ≥16, ≥20, and ≥24 mM units/mL, respectively).

Secondary analyses evaluated the potential effect of other variables on antibody response to the HPV vaccine, such as age, gender, race, eGFR and use of immunosuppression medications. Within the transplanted group, donor type (living vs. deceased), number of transplants and time from transplant were also evaluated.

In addition, although the sample size was small, data was collected regarding safety of the HPV vaccine in this patient population, since it has not been well-studied. All subjects received a vaccination report card (VRC) after each vaccine dose administration. On the VRC, the parent/guardian was asked to record the subject's oral evening temperature daily for 5 days. In addition, injection-site and systemic adverse events (AEs) for a total of 15 days after each vaccination were recorded by parent/guardian. For injection-site erythema and swelling, subjects were instructed by the VRC to measure an injection-site reaction at its greatest width (“maximum size”) from edge to edge in maximum units ranging from 0 to >7 inches (17.5 cm) on the VRC, rounding up to the next unit if in between 2 units [each unit on the VRC measured ~1 inch (2.5 cm)]. For all AEs, subjects were instructed by the VRC to estimate the severity of AEs as mild (awareness of symptom but easily tolerated), moderate (discomfort enough to cause interference with usual activities), or severe (incapacitating with inability to work or do usual activity). Serious AEs were collected for the whole duration of the study regardless of causality and were followed for outcome. In addition, for KT recipients, data was specifically collected on any biopsy-proven acute rejection episodes after T1 up to 6 months after the last dose of the vaccine administration.

### Statistical Analyses

Basic demographic and clinical characteristics are presented as means [standard deviations (SD)] for age, median [interquartile range (IQR)] for eGFR due to non-linearity, and frequencies (percentages) for categorical variables (sex, race, use of immunosuppression medications), with analyses comparing groups performed with ANOVA, Kruskal–Wallis, and Chi–Square tests, respectively. The two main outcomes of serotiter and seroconversion were assessed to determine if there were significant differences between groups at T2. Serotiter values were analyzed using Kruskal–Wallis non-parametric tests and seroconversion proportions using Fisher's Exact tests, with descriptive statistics being given as medians (IQRs) and frequencies (percentages), respectively. Pairwise comparisons were made for serotiter levels using a Bonferroni adjusted *p*-0.0167 to control for inflated type I error rates. Similar analyses, for demographics and the two outcome variables, were also performed comparing those who were on immunosuppression medications vs. those who were not, with similar analyses, using Student's *t*-tests, Wilcoxon rank-sum tests, and Fisher's Exact tests. All analyses were performed using SAS v9.4 (SAS Institute, Cary, NC) and all analytic assumptions were verified. Non-inferiority analyses were also performed, but all were non-significant due to the extremely low power due to our small sample size (results not presented).

## Results

A total of 72 subjects were enrolled: 28 from Riley Hospital for Children in Indianapolis; 15 from Primary Children's Hospital in Salt Lake City; 9 from University of Minnesota Masonic Children's Hospital in Minneapolis; 9 from Medical University of South Carolina Children's Hospital in Charleston; 5 from Kentucky Children's Hospital in Lexington; 5 from C.S. Mott Children's Hospital in Ann Arbor; 1 from Nationwide Children's Hospital in Columbus. A total of 65 participants completed the study: 18 participants in the CKD group, 18 participants in the dialysis group and 29 participants in the KT group (7 subjects were excluded for either not receiving all three doses of the vaccine or for not doing both serology collections). The average age of enrollment was 13.6 years (SD 2.6) and little over one half were males (*N* = 34, 54.8%). The majority of participants were white (*N* = 41, 65.1%) or black (*N* = 11, 17.5%). Table 1 compares the demographics of the three groups with differences demonstrated among the three groups in regards to sex, race, eGFR and use of immunosuppression medications. All the KT patients were taking immunosuppression medications; in addition, some of the patients in the other two groups were also taking immunosuppression medications to treat the immune-mediated diseases leading to CKD or dialysis). Within the KT group (*n* = 29) the median (range) time since first transplant was 3.9 years (3.6, 10.5), about half (51.9%) had received a kidney from a living donor, one individual had had two transplants; two KT recipients reported events of rejection during the study enrollment.

**Table 1:** Demographics and renal status at enrollment^*^.

	**Overall (*n* = 65)**	**CKD (*n* = 18)**	**Dialysis (*n* = 18)**	**Transplant (*n* = 29)**	*p*-value^**^
Age [years; mean (SD)]	13.6 (2.6)	12.9 (2.7)	14.9 (2.5)	13.2 (2.4)	0.055
Sex [Male; *N* (%)]	34 (54.8)	12 (70.6)	4 (25.0)	18 (62.1)	0.018
Race [*N* (%)]					
Asian	2 (3.2)	0 (0)	2 (12.5)	0 (0)	0.006
Black	11 (17.5)	2 (11.1)	2 (12.5)	7 (24.1)	
Hispanic	9 (14.3)	2 (11.1)	6 (37.5)	1 (3.5)	
White	41 (65.1)	14 (77.8)	6 (37.5)	21 (72.4)	
Use of immunosuppression medications [*N* (%)]	38 (61.3)	1 (5.9)	8 (50.0)	29 (100)	<0.001
eGFR [mL/min/1.73 m^2^] [median (IQR)]	31.6 (12.8, 61.9)	30.0 (20.6, 31.6)	9.6 (6.6, 11.2)	63.1 (45.7, 85.6)	<0.001

* Values are means (standard deviation) for age, frequencies (percentages) for categorical variables, and median (IQR) for eGFR;

** *p-values are from ANOVA, Fisher's Exact, and Kruskal–Wallis tests, respectively. Frequencies may not add to column totals due to missing data*.

All participants were seronegative for all four of the vaccine HPV types at enrollment, except for three participants who were not naïve for HPV 6, and thus excluded from serotiter and seroconversion analyses for that particular HPV type only. Serotiters for all HPV types were detected post-vaccination at T2, with KT patients having significantly lower GMTs for all serotypes; pairwise comparisons indicated the significant associations were driven by the difference between CKD and KT groups, although CKD and dialysis groups were also different for HPV type 11 (Table 2). Examination of the seroconversion rates based on manufacturer cutoffs showed significant heterogeneity between the groups (Table 3). The seroconversion rates post-vaccination were significantly lower for the transplanted group for three of the serotypes (HPV 6, 11, and 18). Although not statistically different, seroconversion rates were also lower for the HPV 16 serotype.

**Table 2:** Antibody responses to HPV vaccine (GMTs)—continuous variable by groups^*^.

	**CKD (*n* = 18)**	**Dialysis (*n* = 18)**	**Transplant (*n* = 29)**	*p*-value^**^
HPV 6^#^	869 (128, 1739); 539.67	217.5 (137, 326); 205.37	115 (12, 590); 113.16	0.016
HPV 11	1694.5 (622, 2740); 1208.30	431.5 (180, 996); 370.08	83 (13, 675); 110.13	0.001
HPV 16	5639.5 (934, 9189); 4390.79	1581.5 (436, 3404); 1709.62	436 (74, 4316); 508.43	0.011
HPV 18	1406.5 (150, 5121); 1039.62	331.5 (69, 622); 266.45	52 (9, 497); 91.30	0.004

* Values are medians (IQRs); mean GMTs at T2.

** p-values are from Kruskal–Wallis non-parametric tests.

# *HPV 6 analyses had sample sizes of 17, 16 and 29, respectively, as those who were not naïve at baseline were excluded for that particular outcome's analysis. Post-hoc pairwise comparisons were made with Wilcoxon rank-sum tests, using a p-value of 0.017 for significance to control for inflated type I error rate; pairwise comparisons indicate the significant associations are driven by the difference between CKD and KT groups, although CKD and dialysis groups were also different for HPV type 11*.

**Table 3:** Seroconversion rates—categorical variable by groups^*^.

	**CKD** **(*n* = 18)**	**Dialysis** **(*n* = 18)**	**Transplant** **(*n* = 29)**	*p*-value^**^
HPV 6^#^	17 (100)	15 (93.8)	21 (72.4)	0.017
HPV 11	18 (100)	16 (88.9)	20 (69.0)	0.010
HPV 16	18 (100)	17 (94.4)	26 (89.7)	0.570
HPV 18	17 (94.4)	15 (83.3)	18 (62.1)	0.032

* Values are frequencies (percentages) of participants who seroconverted, defined as being HPV naïve at T1 and having GMTs ≥20, ≥16, ≥20 and ≥24 milli-Merck units/mL for HPV types 6, 11, 16, and 18, respectively at T2.

** p-values are from Fisher's Exact tests.

# *HPV 6 analyses had sample sizes of 17, 16, and 29, respectively as those who were not naïve at baseline were excluded for that particular outcome's analysis*.

All variables found to be different among the three treatment groups were examined directly with the two outcome variables, GMTs at T2 and seroconversion. Age, sex, race, and eGFR were found to have no association with GMTs at T2 or actual seroconversion. However, when only the KT population was examined, being a female KT-recipient was associated with greater GMTs at T2 for HPV 6 and 11 only (*p* = 0.020 and 0.046, respectively) and with higher seroconversion rates for HPV 18 only (90.9 vs. 44.4% for females and males, respectively; *p* = 0.020). Although statistically significant, this is difficult to interpret clinically with the inconsistencies among the various HPV types and small numbers for multiple analyses.

Comparing subjects who were using immunosuppression medications (including KT recipients, as well as CKD 3, 4, and 5 on immunosuppression medications to treat their primary kidney conditions) vs. not, the immunosuppression medication-using group had significantly lower serotiters for all four HPV serotypes at T2 and lower seroconversion rates for HPV 6, 11, and 18 (Table 4). The immunosuppression medications used in the transplant population included prednisone, tacrolimus, cyclosporine A and mycophenolate mofetil. The immunosuppression medications used to treat the primary kidney diseases in the CKD and dialysis patients included prednisone, tacrolimus, mycophenolate mofetil and azathioprine. Unlike in the KT population, when the male vs. female differences were examined specifically in the immunosuppression medication-using group, no differences were seen.

**Table 4:** Comparison between subjects who used immunosuppression medications vs. subjects not on immunosuppression medications^*^.

	**Immunosuppression medications use (*n* = 38)**	**No Immunosuppression medication use (*n* = 24)**	*p*-value^**^
Age [years; mean (SD)]	13.5 (2.4)	13.6 (3.1)	0.904
Sex [Male; *N* (%)]	19 (50.0)	14 (60.9)	0.440
Race [*N* (%)]			
Asian	1 (2.6)	1 (4.2)	0.030
Black	9 (23.7)	2 (8.3)	
Hispanic	2 (5.3)	7 (29.2)	
White	26 (68.4)	14 (58.3)	
eGFR [mL/min/1.73 m^2^] [median (IQR)]	56.7 (20.4, 69.4)	21.4 (9.8, 31.1)	<0.001
GMTs at T2 [median (IQR)]			
HPV 6^#^	120 (19, 564)	362.5 (140.5, 1216)	0.005
HPV 11	121 (13, 675)	1247 (522, 2028.5)	<0.001
HPV 16	436 (89, 3404)	4751.5 (1829, 9091)	<0.001
HPV 18	50 (9, 497)	963.5 (396, 4243.5)	<0.001
Seroconversion [*N* (%)]			
HPV 6^#^	26 (74.3)	24 (100)	0.008
HPV 11	27 (71.1)	24 (100)	0.004
HPV 16	34 (89.5)	24 (100)	0.151
HPV 18	24 (63.2)	23 (95.8)	0.005

* Values are means (standard deviation) for age; frequencies (percentages) for sex, race, and seroconversion; median (IQR) for eGFR and for GMTs.

** p-values are from Student's t-test, Fisher's Exact tests, and Wilcoxon tests, respectively. Frequencies may not add to column totals due to missing data. The total number of subjects in this analysis was 38 + 24 = 62, as opposed to 65 subjects in the other analyses. This is due to missing medication information from the data collection forms on three subjects.

# *HPV 6 analyses had sample sizes of 35 and 24, respectively, as those who were not naïve at baseline were excluded for that particular outcome's analysis*.

Within the transplant population, neither the donor type, neither time since transplant, nor the number of transplants, made any difference in terms of GMTs at T2 or seroconversion rates.

As for the AEs, they were minimal, limited to only mild local reactions. Two patients underwent acute rejection episodes within 6 months following the last dose of the vaccine. However, upon review of chart data, these events were considered to be likely related to medication non-adherence.

## Discussion

American Society of Transplantation recommends that HPV vaccine be given to all SOT candidates and recipients in the recommended age-group (27). However, the efficacy and tolerability of the HPV vaccine in such populations, as well as the optimal timing to do so, are not completely understood. Our results demonstrated that the HPV vaccine was well-tolerated and an adequate immune response for all four HPV types was observed with CKD and dialysis patients. However, the immune response was slightly lower within the transplanted population and immunosuppressant medications had an effect on lack of seroconversion. HPV vaccine acceptance by patients and their parents/guardians has always been problematic even in the general population (29, 30) and probably more so in these specific groups, where data is scant (26). Our study provides further evidence that patients who are likely to need a KT in the future respond best to the HPV vaccine pre-transplantation, while with CKD or on dialysis. As the majority of causes of end-stage renal disease in pediatrics are congenital diseases in which the kidney function slowly deteriorates over time, pediatric nephrologists often have the advantage of being able to better plan a KT (whether preemptive or not) considering multiple aspects, including vaccinations, compared to their adult nephrologist colleagues (26). This study showed that girls and boys with CKD and on dialysis had stronger immunologic responses to the HPV vaccine compared to those who were already transplanted. Therefore, this study should help pediatric nephrologists and pediatricians in their work with patients and families to pursue and complete the administration of HPV vaccine ideally prior to transplant whenever possible, but also post-transplant in cases where it was not given prior to KT. Is is to be noted that in 2015 the Advisory Committee on Immunization Practices (ACIP) recommended the 9-valent HPV vaccine (9vHPV) (Gardasil 9, Merck and Co., Inc.) as an alternative to the quadrivalent vaccine (31). The 9-valent vaccine was found to be safe, provided more extensive serotype coverage and it was even found to be cost-saving when compared with the quadrivalent vaccine, therefore it is currently more widely used (31). Our study was initiated well before 2015, hence the use of the quadrivalent vaccine.

It is important for the HPV vaccine to be given before HPV infection is acquired, meaning before the onset of sexual activity (28). This is the rationale behind the fact that the HPV vaccine is recommended to be given at 11–12 years of age, although it can be administered starting at age 9 (28). In the US, the proportion of males who reported having sexual intercourse before age 13 years varied from 5 to 25% across metropolitan sites (32), hence the need to vaccinate at a young age, designed to capture most teenagers before becoming sexually active. In our study, the onset of sexual activity was not assessed out of concerns that in a multisite trial, the investigators could not assure patient confidentiality relative to their parents/caregivers for all participants. However, obtaining this information in future studies would be helpful in assessing clinical risk and outcomes in patients with chronic diseases (33, 34). In children with CKD and on dialysis, contrary to what was believed in the past, recent cohort studies have shown that growth and sexual maturation are only slightly delayed compared to healthy subjects (35). Although there is lack of specific data, given the circumstances, it is plausible that adolescents with CKD or on dialysis are not far behind from their peers in terms of their onset of sexual activity. It is therefore of concern that in our study the mean age of subjects was 13.6 (SD 2.6), meaning that our patient population was vaccinated on average later than the recommended age.

Our study showed for the first time both in boys and girls that overall, transplant patients had a decreased response to the HPV vaccine compared to CKD and dialysis patients, likely related to their state of medication-induced immunosuppression. However, specifically for HPV 16, seroconversion rates were not found to be significantly lower for KT patients and neither were they found to be lower for patients taking immunosuppression medications. Although power may have biased this result, it is also somewhat reassuring since the majority of HPV cancers are associated with type 16. At the very least, the data supports ongoing vaccination in this group due to the fact that the majority of individuals did effectively respond to the vaccine. Review of literature revealed that the first studies to look into HPV vaccine responses in immunosuppressed populations was in HIV-positive cohorts, which showed promising results, as 95–100% of the subjects demonstrated seroconversion (36–38). Thus, seroconversion was not much lower than in healthy populations (39). Surprisingly, the first study of the immunogenicity of the HPV vaccine in SOT recipients was done in a cohort of 47 adults (females and males) and reported much lower rates of seroconversion: 63.2, 68.4, 63.2, and 52.6% for HPV 6, 11, 16, and 18, respectively (40). Somewhat similarly, in pediatrics, a study of a cohort of 57 patients (females only) aged 9–21 years old with CKD, on dialysis, or post-KT, showed that among patients with transplants, the percentages achieving seropositivity were significantly lower compared to CKD and dialysis patients: 63.6, 63.6, and 72.7% for HPV 6, 11, and 18, respectively (41). Our study included both girls and boys age 10 to 16 with CKD, on dialysis, or post-KT and showed lower seroconversion rates: 72.4, 69.0, and 62.1% for HPV 6, 11, and 18, respectively, in transplant patients, thus demonstrating similar results for the first time in boys, as well.

In this study, it is likely that the entire population was affected by varying levels of immunosuppression, regardless of the study group. Indeed, patients with CKD and more so the ones on dialysis have been shown to have various degrees of immunosuppression, whether caused by defects in neutrophil function, antigen processing, cell-mediated immunity, or antibody-mediated immunity (42, 43). Antibody responses to certain vaccines have been found to be attenuated in this patient population for certain vaccines, such as the measles, mumps and rubella (44). On the contrary, for other vaccines immune responses have been found to be similar to the general population (45). In the case of HPV vaccine, a study on pediatric and adult patients with CKD 4, 5, and on dialysis showed 98.2, 100, 100, and 98.2% serocoversion for HPV genotypes 6, 11, 16, and 18, respectively (46). These values are comparable to the data generated in healthy adult women large populations, which reported 99.7, 99.5, 99.7, and 99.2% seropositivity for HPV genotypes 6, 11, 16, and 18, respectively (39). Our study also reported robust immune responses in the CKD and dialysis pediatric patients. Although there was a non-statistically significant tendency to less robust responses for dialysis patients compared to CKD, this was probably explained by the fact that half of the dialysis patients were on immunosuppressive medications for their kidney conditions. In addition, we have found no difference in terms of HPV vaccine responses between patients on hemodialysis vs. peritoneal dialysis in our study population (data not presented). It is likely that being on anti-rejection medications creates a more profound state of immunosuppression in transplant recipients than the immune dysregulation seen in CKD or while on dialysis, since KT patients had less robust immune responses to the HPV vaccine in our study. Likewise, patients with CKD or on dialysis who were on immunosuppression medications for their primary kidney diseases, which are in fact similar to anti-rejection medications, also demonstrated poorer seroconversion rates.

It is possible that girls and boys who are KT recipients respond differently to the HPV vaccine. The quadrivalent HPV vaccine was approved in the US in 2006 for use in girls/women only. Subsequently, in 2009, the indication was extended to boys/men as well. The few studies that compared the seroconversion rates to HPV vaccine between males and females in the general population have not shown any differences (47, 48). Our study showed that overall there were no differences between males and females in terms of HPV antibody responses. However, when data was analyzed specifically for the KT recipients, male subjects had lower GMTs at T2 for HPV 6 and 11 (but not 16 or 18) and lower seroconversion rates for HPV 18 (but not for HPV6, HPV11, or HPV16) compared to females. Although statistically significant, this is difficult to interpret clinically with the inconsistencies among the various HPV types and small numbers for multiple analyses. Interestingly enough, unlike in the KT population, when the male vs. female differences were examined specifically in the immunosuppression medication-using group, no differences were seen. Sex-based differences in immune function and responses to vaccination have been described before, where females typically developed higher antibody responses and experienced more adverse reactions following vaccination than males for bacillus Calmette-Guerin, measles, mumps, rubella, yellow fever and influenza vaccines (49, 50). In addition, men SOT recipients have a higher incidence of malignancies, also suggesting the fact that SOT men could become more immunosuppressed than women when treated with anti-rejection medications (51). In any event, the fact that male KT recipients tend to have lower antibody titers and seroconversion rates for certain serotypes following HPV vaccination, while of uncertain significance, still raises questions about optimal vaccination strategies specifically in this patient population, which has been already shown to also have lower vaccination rates compared to females (52).

AEs reported were similar to those noted in prior studies, limited to minor vaccine local skin reactions. Two patients underwent acute rejection episodes within 6 months following dose 3 of the HPV vaccine. In 2014, a study initiated HPV vaccination in 14 pediatric KT recipients; unfortunately six of them developed acute rejection shortly after the initiation of the vaccine series, which lead to an early termination of the study (53). In contrast, in 2016, only two of the 23 pediatric KT recipients of HPV vaccination were reported to have acute rejection episodes (41). In our patient population, we report a similar acute rejection burden; upon chart review, it was felt that our two cases of acute rejection were related to medication non-adherence, as opposed to vaccine administration.

We acknowledge that our study has certain limitations. Like in many pediatric SOT studies, our study was limited by its small sample size. Unfortunately three subjects were missing information on medication use in the data collection form and some were not complete, thus impairing our ability to analyze the effect of specific immunosuppresion medications. Indeed, for specific vaccinations, for example influenza vaccination in KT recipients, it has been demonstrated that different imunosuppression regimens can influence the immune response (54). In addition, our study lacked long-term follow up, as we did not look into long-term HPV-vaccine induced antibody persistence post-transplantation; other studies suggested that there was a decline in titers over time (40). Lastly, we were unable to determine factors beyond immunosuppression that may play a role in preventing seroconversion in transplant recipients. However, this is the largest and the only multicenter study that examined the humoral response to the HPV vaccine in both girls and boys with KTs, compared to CKD and on dialysis. It is also the first study that compared the humoral response between girls and boys in these populations. In addition, we included only data for the subjects who were HPV naïve at baseline, had complete data in terms of antibody titers and completed the entire three dose vaccination series.

In conclusion, this study addresses the immune response to the HPV vaccine in pediatric KT recipients, CKD and dialysis patients, as well as its safety. The HPV vaccine was well-tolerated in these populations. Pediatric KT recipients had in general lower GMTs and seroconversion rates compared to their peers in the CKD and dialysis groups. In addition, immunosuppression (either due to the need to address immune-mediated diseases of the native kidneys or to prevent rejection in KT) played a role in the lack of seroconversion. Our results emphasize the importance of advocating for HPV vaccination prior to KT and acknowledge its safety post transplantation. However, questions still remain. Specifically, studies to identify factors associated with the lack of seroconversion in KT recipients are needed for targeted interventions. In addition, future studies should investigate the effect of a supplemental dose of HPV vaccine in KT recipients who do not seroconvert. It would also be important to evaluate the long-term persistence of antibodies post-KT and the possible need for re-immunization post-KT.

## Data Availability Statement

All datasets generated for this study are included in the article/supplementary material.

## Ethics Statement

The studies involving human participants were reviewed and approved by Indiana University Office of Research Administration Institutional Board Review. Written informed consent to participate in this study was provided by the participants' legal guardian/next of kin.

## Author Contributions

CN generated the hypothesis, obtained funding, designed the study, recruited subjects at her institution, interpreted data, and wrote the manuscript. RN, PV, KT, AC, MM, and JM had solutions for improving design, recruited subjects at their respective institutions, and corrected the manuscript. JS analyzed and presented data, while also helping CN and MS write the statistics paragraph and interpret data. MS mentored and helped CN in all of the above endeavors.

### Conflict of Interest

The authors declare that the research was conducted in the absence of any commercial or financial relationships that could be construed as a potential conflict of interest.

## Abbreviations

SOT: solid organ transplant
KT: kidney transplant
HPV: human papilloma virus
CKD: chronic kidney disease
GFR: glomerular filtration rate
eGFR: estimated GFR calculated using the modified Schwartz formula (0.413 × height in cm/serum creatinine in mg/dL)
T1: time 1 (day 1 of enrollment prior to dose 1 of the vaccine administration)
T2: time 2 (one month after vaccine series was completed)
VRC: vaccination report card
AEs: adverse events
SD: standard deviation
IQR: interquartile range.

## References

[1] Kitchlu A Dixon S Dirk JS Chanchlani R Vasilevska-Ristovska J Borges K. Elevated risk of cancer after solid organ transplant in childhood: a population-based cohort study. Transplantation. (2019) 103:588–96. 10.1097/TP.0000000000002378 30048393

[2] Yanik EL Smith JM Shiels MS Clarke CA Lynch CF Kahn AR. Cancer risk after pediatric solid organ transplantation. Pediatrics. (2017) 139:e20163893. 10.1542/peds.2016-3893 28557749 PMC5404730

[3] Hall EC Pfeiffer RM Segev DL Engels EA. Cumulative incidence of cancer after solid organ transplantation. Cancer. (2013) 119:2300–8. 10.1002/cncr.28043 23559438 PMC4241498

[4] D'Arcy ME Coghill AE Lynch CF Koch LA Li J Pawlish KS. Survival after a cancer diagnosis among solid organ transplant recipients in the United States. Cancer. (2019) 125:933–42. 10.1002/cncr.31782 30624768 PMC6403005

[5] Smith JM Martz K Blydt-Hansen TD. Pediatric kidney transplant practice patterns and outcome benchmarks, 1987-2010: a report of the North American pediatric renal trials and collaborative studies. Pediatr Transplant. (2013) 17:149–57. 10.1111/petr.12034 23281637

[6] Awan AA Niu J Pan JS Erickson KF Mandayam S Winkelmayer WC. Trends in the causes of death among kidney transplant recipients in the United States (1996-2014). Am J Nephrol. (2018) 48:472–81. 10.1159/000495081 30472701 PMC6347016

[7] Hinten F Meeuwis KA van Rossum MM de Hullu JA. HPV-related (pre)malignancies of the female anogenital tract in renal transplant recipients. Crit Rev Oncol Hematol. (2012) 84:161–80. 10.1016/j.critrevonc.2012.02.008 22425015

[8] Liao JB Fisher CE Madeleine MM. Gynecologic cancers and solid organ transplantation. Am J Transplant. (2019) 19:1266–77. 10.1111/ajt.15292 30725527

[9] Madeleine MM Finch JL Lynch CF Goodman MT Engels EA. HPV-related cancers after solid organ transplantation in the United States. Am J Transplant. (2013) 13:3202–9. 10.1111/ajt.12472 24119294 PMC4049182

[10] Smith JS Lindsay L Hoots B Keys J Franceschi S Winer R. Human papillomavirus type distribution in invasive cervical cancer and high-grade cervical lesions: a meta-analysis update. Int J Cancer. (2007) 121:621–32. 10.1002/ijc.22527 17405118

[11] Antonsson A Neale RE Boros S Lampe G Coman WB Pryor DI. Human papillomavirus status and p16(INK4A) expression in patients with mucosal squamous cell carcinoma of the head and neck in Queensland, Australia. Cancer Epidemiol. (2015) 39:174–81. 10.1016/j.canep.2015.01.010 25677091

[12] Satterwhite CL Torrone E Meites E Dunne EF Mahajan R Ocfemia MC. Sexually transmitted infections among US women and men: prevalence and incidence estimates, 2008. Sex Transm Dis. (2013) 40:187–93. 10.1097/OLQ.0b013e318286bb53 23403598

[13] Komlos KF Kocjan BJ Kosorok P Luzar B Meglic L Potocnik M. Tumor-specific and gender-specific pre-vaccination distribution of human papillomavirus types 6 and 11 in anogenital warts and laryngeal papillomas: a study on 574 tissue specimens. J Med Virol. (2012) 84:1233–41. 10.1002/jmv.23318 22711351

[14] Orlando G Bianchi S Fasolo MM Mazza F Frati ER Rizzardini G. Cervical Human Papillomavirus genotypes in HIV-infected women: a cross-sectional analysis of the VALHIDATE study. J Prev Med Hyg. (2017) 58:E259–e65. 10.15167/2421-4248/jpmh2017.58.4.804 29707656 PMC5912788

[15] Long ME Chantigian PDM Weaver AL. Cervical cytology and histology after solid organ transplant: a longitudinal cohort study. J Low Genit Tract Dis. (2018) 22:362–6. 10.1097/LGT.0000000000000414 29975335

[16] Veroux M Corona D Scalia G Garozzo V Gagliano M Giuffrida G. Surveillance of human papilloma virus infection and cervical cancer in kidney transplant recipients: preliminary data. Transplant Proc. (2009) 41:1191–4. 10.1016/j.transproceed.2009.03.015 19460514

[17] Berkhout RJ Bouwes Bavinck JN ter Schegget J. Persistence of human papillomavirus DNA in benign and (pre)malignant skin lesions from renal transplant recipients. J Clin Microbiol. (2000) 38:2087–96. 10834958 10.1128/jcm.38.6.2087-2096.2000 PMC86734

[18] Pietrzak B Mazanowska N Ekiel AM Durlik M Martirosian G Wielgos M. Prevalence of high-risk human papillomavirus cervical infection in female kidney graft recipients: an observational study. Virol J. (2012) 9:117. 10.1186/1743-422X-9-117 22709394 PMC3489881

[19] Meeuwis KA Melchers WJ Bouten H van de Kerkhof PC Hinten F Quint WG. Anogenital malignancies in women after renal transplantation over 40 years in a single center. Transplantation. (2012) 93:914–22. 10.1097/TP.0b013e318249b13d 22377788

[20] Busnach G Piselli P Arbustini E Baccarani U Burra P Carrieri MP. Immunosuppression and cancer: a comparison of risks in recipients of organ transplants and in HIV-positive individuals. Transplant Proc. (2006) 38:3533–5. 10.1016/j.transproceed.2006.10.144 17175324

[21] Guerra FM Rosella LC Dunn S Wilson SE Chen C Deeks SL. Early impact of Ontario's human papillomavirus (HPV) vaccination program on anogenital warts (AGWs): A population-based assessment. Vaccine. (2016) 34:4678–83. 10.1016/j.vaccine.2016.08.020 27527815

[22] Niccolai LM Meek JI Brackney M Hadler JL Sosa LE Weinberger DM. Declines in Human Papillomavirus (HPV)-associated high-grade cervical lesions after introduction of HPV vaccines in connecticut, United States, 2008-2015. Clin Infect Dis. (2017) 65:884–9. 10.1093/cid/cix455 28520854

[23] Arbyn M Xu L. Efficacy and safety of prophylactic HPV vaccines. a Cochrane review of randomized trials. Expert Rev Vaccines. (2018) 17:1085–91. 10.1080/14760584.2018.1548282 30495978

[24] Collins S Mazloomzadeh S Winter H Blomfield P Bailey A Young LS. High incidence of cervical human papillomavirus infection in women during their first sexual relationship. BJOG. (2002) 109:96–8. 10.1111/j.1471-0528.2002.01053.x 11845815

[25] Castellino SM Allen KE Pleasant K Keyes G Poehling KA Tooze JA. Suboptimal uptake of human papillomavirus (HPV) vaccine in survivors of childhood and adolescent and young adult (AYA) cancer. J Cancer Surviv. (2019) 13:730–8. 10.1007/s11764-019-00791-9 31342304 PMC6832771

[26] Fox TG Nailescu C. Vaccinations in pediatric kidney transplant recipients. Pediatr Nephrol. (2019) 34:579–91. 10.1007/s00467-018-3953-z 29671067

[27] Danziger-Isakov L Kumar D. Vaccination in solid organ transplantation. Am J Transplant. (2013) 13 (Suppl. 4):311–7. 10.1111/ajt.12122 23465023

[28] Centers for Disease Control and Prevention (CDC). Recommendations on the use of quadrivalent human papillomavirus vaccine in males–Advisory Committee on Immunization Practices (ACIP), 2011. MMWR Morb Mortal Wkly Rep. (2011) 60:1705–8. 22189893

[29] Dempsey AF Patel DA. HPV vaccine acceptance, utilization and expected impacts in the U.S.: Where are we now? Hum Vaccin. (2010) 6:715–20. 10.4161/hv.6.9.12730 20855941 PMC3010327

[30] Donahue KL Stupiansky NW Alexander AB Zimet GD. Acceptability of the human papillomavirus vaccine and reasons for non-vaccination among parents of adolescent sons. Vaccine. (2014) 32:3883–5. 10.1016/j.vaccine.2014.05.035 24844150

[31] Petrosky E Bocchini JA Jr Hariri S Chesson H Curtis CR Saraiya M. Use of 9-valent human papillomavirus (HPV) vaccine: updated HPV vaccination recommendations of the advisory committee on immunization practices. MMWR Morb Mortal Wkly Rep. (2015) 64:300–4. 25811679 PMC4584883

[32] Lindberg LD Maddow-Zimet I Marcell AV. Prevalence of sexual initiation before Age 13 years among male adolescents and young adults in the United States. JAMA Pediatr. 2019. 10.1001/jamapediatrics.2019.0458 30958512 PMC6547075

[33] Gambadauro P Carli V Hadlaczky G Sarchiapone M Apter A Balazs J. Correlates of sexual initiation among European adolescents. PloS ONE. (2018) 13:e0191451. 10.1371/journal.pone.0191451 29420612 PMC5805230

[34] Kahn NF Halpern CT. Associations between patterns of sexual initiation, sexual partnering, and sexual health outcomes from adolescence to early adulthood. Arch Sex Behav. (2018) 47:1791–810. 10.1007/s10508-018-1176-9 29594701 PMC6501817

[35] Franke D Winkel S Gellermann J Querfeld U Pape L Ehrich JH. Growth and maturation improvement in children on renal replacement therapy over the past 20 years. Pediatr Nephrol. (2013) 28:2043–51. 10.1007/s00467-013-2502-z 23708760

[36] Wilkin T Lee JY Lensing SY Stier EA Goldstone SE Berry JM. Safety and immunogenicity of the quadrivalent human papillomavirus vaccine in HIV-1-infected men. J Infect Dis. (2010) 202:1246–53. 10.1086/656320 20812850 PMC3118428

[37] Levin MJ Moscicki AB Song LY Fenton T Meyer WA 3rd Read JS. Safety and immunogenicity of a quadrivalent human papillomavirus (types 6, 11, 16, and 18) vaccine in HIV-infected children 7 to 12 years old. J Acquir Immune Defic Syndr. (2010) 55:197–204. 10.1097/QAI.0b013e3181de8d26 20574412 PMC3033215

[38] Weinberg A Song LY Saah A Brown M Moscicki AB Meyer WA 3rd. Humoral, mucosal, and cell-mediated immunity against vaccine and nonvaccine genotypes after administration of quadrivalent human papillomavirus vaccine to HIV-infected children. J Infect Dis. (2012) 206:1309–18. 10.1093/infdis/jis489 22859825 PMC3529604

[39] Brown DR Garland SM Ferris DG Joura E Steben M James M. The humoral response to Gardasil over four years as defined by total IgG and competitive Luminex immunoassay. Hum Vaccin. (2011) 7:230–8. 10.4161/hv.7.2.13948 21307649

[40] Kumar D Unger ER Panicker G Medvedev P Wilson L Humar A. Immunogenicity of quadrivalent human papillomavirus vaccine in organ transplant recipients. Am J Transplant. (2013) 13:2411–7. 10.1111/ajt.12329 23837399 PMC4583130

[41] Nelson DR Neu AM Abraham A Amaral S Batisky D Fadrowski JJ. Immunogenicity of human papillomavirus recombinant vaccine in children with CKD. Clin J Am Soc Nephrol. (2016) 11:776–84. 10.2215/CJN.09690915 27055465 PMC4858485

[42] Chonchol M. Neutrophil dysfunction and infection risk in end-stage renal disease. Semin Dial. (2006) 19:291–6. 10.1111/j.1525-139X.2006.00175.x 16893406

[43] Dalrymple LS Go AS. Epidemiology of acute infections among patients with chronic kidney disease. Clin J Am Soc Nephrol. (2008) 3:1487–93. 10.2215/CJN.01290308 18650409 PMC4571152

[44] Schulman SL Deforest A Kaiser BA Polinsky MS Baluarte HJ. Response to measles-mumps-rubella vaccine in children on dialysis. Pediatr Nephrol. (1992) 6:187–9. 10.1007/BF00866312 1571219

[45] Fadrowski JJ Furth SL. Varicella zoster virus: vaccination and implications in children with renal failure. Expert Rev Vaccines. (2004) 3:291–8. 10.1586/14760584.3.3.291 15176945

[46] Praditpornsilpa K Kingwatanakul P Deekajorndej T Rianthavorn P Susantitaphong P Katavetin P. Immunogenicity and safety of quadrivalent human papillomavirus types 6/11/16/18 recombinant vaccine in chronic kidney disease stage IV, V and VD. Nephrol Dial Transplant. (2017) 32:132–6. 10.1093/ndt/gfv444 26932687

[47] Block SL Nolan T Sattler C Barr E Giacoletti KE Marchant CD. Comparison of the immunogenicity and reactogenicity of a prophylactic quadrivalent human papillomavirus (types 6, 11, 16, and 18) L1 virus-like particle vaccine in male and female adolescents and young adult women. Pediatrics. (2006) 118:2135–45. 10.1542/peds.2006-0461 17079588

[48] Ferris D Samakoses R Block SL Lazcano-Ponce E Restrepo JA Reisinger KS. Long-term study of a quadrivalent human papillomavirus vaccine. Pediatrics. (2014) 134:e657–65. 10.1542/peds.2013-4144 25136050

[49] Klein SL Marriott I Fish EN. Sex-based differences in immune function and responses to vaccination. Trans R Soc Trop Med Hyg. (2015) 109:9–15. 10.1093/trstmh/tru167 25573105 PMC4447843

[50] Furman D Hejblum BP Simon N Jojic V Dekker CL Thiebaut R. Systems analysis of sex differences reveals an immunosuppressive role for testosterone in the response to influenza vaccination. Proc Natl Acad Sci USA. (2014) 111:869–74. 10.1073/pnas.1321060111 24367114 PMC3896147

[51] Bhat M Mara K Dierkhising R Watt KDS. Immunosuppression, race, and donor-related risk factors affect de novo cancer incidence across solid organ transplant recipients. Mayo Clin Proc. (2018) 93:1236–46. 10.1016/j.mayocp.2018.04.025 30064826

[52] Choi Y Eworuke E Segal R. What explains the different rates of human papillomavirus vaccination among adolescent males and females in the United States? Papillomavirus Res. (2016) 2:46–51. 10.1016/j.pvr.2016.02.001 29074185 PMC5886892

[53] Gomez-Lobo V Whyte T Kaufman S Torres C Moudgil A. Immunogenicity of a prophylactic quadrivalent human papillomavirus L1 virus-like particle vaccine in male and female adolescent transplant recipients. Pediatr Transplant. (2014) 18:310–5. 10.1111/petr.12226 24484551

[54] Nailescu C Xu X Zhou H Hall H Wilson AC Leiser JD. Influenza vaccine after pediatric kidney transplant: a Midwest Pediatric Nephrology Consortium study. Pediatr Nephrol. (2011) 26:459–67. 10.1007/s00467-010-1729-1 21181206

